# Capacity Contribution Induced by Pseudo-Capacitance Adsorption Mechanism of Anode Carbonaceous Materials Applied in Potassium-ion Battery

**DOI:** 10.3389/fchem.2019.00640

**Published:** 2019-10-02

**Authors:** Jiahao Liu, Ziqiang Xu, Mengqiang Wu, Yuesheng Wang, Zaghib Karim

**Affiliations:** ^1^School of Materials and Energy, University of Electronic Science and Technology of China, Chengdu, China; ^2^Center of Excellence in Transportation Electrification and Energy Storage, Hydro-Québec, Varennes, QC, Canada

**Keywords:** potassium-ion batteries, carbonaceous anodes, pseudo-capacitance adsorption, surface doping activation, kinetic analysis

## Abstract

The intrinsic bottleneck of graphite intercalation compound mechanism in potassium-ion batteries necessitates the exploitation of novel potassium storage strategies. Hence, utmost efforts have been made to efficiently utilize the extrinsic pseudo-capacitance, which offers facile routes by employing low-cost carbonaceous anodes to improve the performance of electrochemical kinetics, notably facilitating the rate and power characteristics for batteries. This mini-review investigates the methods to maximize the pseudo-capacitance contribution based on the size control and surface activation in recent papers. These methods employ the use of cyclic voltammetry for kinetics analysis, which allows the quantitative determination on the proportion of diffusion-dominated vs. pseudo-capacitance by verifying a representative pseudo-capacitive material of single-walled carbon nanotubes. Synergistically, additional schemes such as establishing matched binder–electrolyte systems are in favor of the ultimate purpose of high-performance industrialized potassium-ion batteries.

## Introduction

Suffering from the geopolitical maldistribution of lithium resources, sodium-ion batteries (SIBs) and potassium-ion batteries (PIBs) reach a hotspot in view of wider resource reserves compared with lithium-ion batteries (LIBs) (2.09 wt% of K vs. 2.36 wt% of Na vs. 0.0017 wt% of Li) (Carmichael, 1989; Larcher and Tarascon, 2015). Significantly, the PIB system has the lowest negative potential (0.15 V below the Li/Li^+^) (Komaba et al., 2015; Eftekhari et al., 2016; Wang et al., 2018a,b) and satisfactory electrochemical kinetics in ionic diffusion kinetics and conductivity theoretically (Okoshi et al., 2013, 2017; Komaba et al., 2015; Eftekhari et al., 2016; Su et al., 2016) in non-aqueous electrolytes, ascribed to low de-solvation due to its weak Lewis acid character (Okoshi et al., 2017; Lei et al., 2018). Similar to the behavior of LIBs in graphite (Wang, 2017), the intercalation mechanism of PIBs involves three potassiation stages, generating the KC_36_ in Stage III, KC_24_ in Stage II, and finally the KC_8_ in Stage I (Jian et al., 2017) with 270 mA h g^−1^, which is far more stable than SIBs (Wang et al., 2013; Zheng et al., 2017). Nevertheless, large Shannon ionic radius (K^+^ = 1.38 Å, Na^+^ = 1.02 Å, Li^+^ = 0.76 Å) and atomic mass (K = 39.10, Na = 22.99, Li = 6.94) (Shannon, 1976) have decreased the theoretical capacity and induced high volume expansion of 61% (Wen et al., 2015; Eftekhari et al., 2016; Zou et al., 2017). Although some strategies enter into consideration such as adopting expand graphite (An et al., 2018), implementing solvent co-intercalation (SCI) (Wang et al., 2013, 2019; David and Singh, 2014), and developing dual-carbon batteries (DCBs) (Carlin et al., 1996, 2010; Beltrop et al., 2017; Fan et al., 2017; Ji et al., 2017), essential kinetics deficiency is hard to surmount.

To address the irreversible expansion induced by equilibrium graphite, amorphous carbons come into the focused sight (Xing et al., 2017), which are admittedly classified as hard carbon (HC) and soft carbon (SC). HC is proven to have a prolonged cycling ability for its randomly oriented bend graphitic layers along the c axis without observed expansion after a thorough potassiation (Jian et al., 2016, 2017). On the contrary, SC is easily graphitized with turbostratic domains although far from the commercial graphite. SC presents obvious expansion to HC if undergoing complete potassiation (Luo et al., 2015a; Wang et al., 2017). Nonetheless, SC has a better rate performance than HC for more aligned domains (Jian et al., 2017), regarded as the reason for the better rate performance.

**Figure 1:**
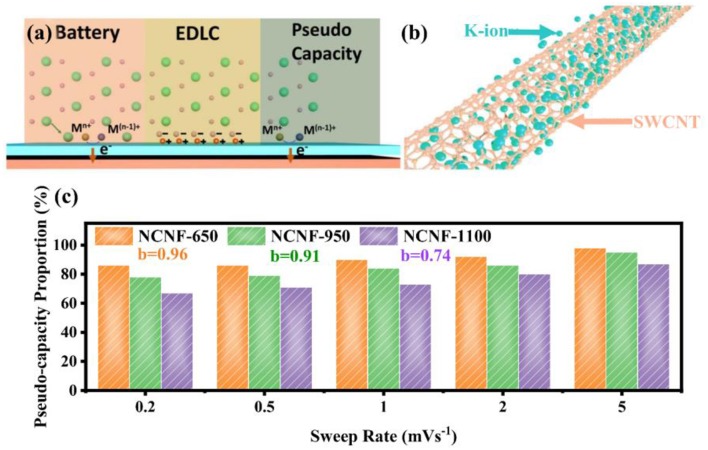
**(a)** Surface processes among battery, EDLC and pseudo-capacitance. **(b)** Diagram of the surface-dominated procedure for SWCNT; with permission from Wiley. **(c)** Pseudo-capacity proportion of NCNF-650, NCNF-950 and NCNF-1100 with *b* value; with permission from Springer.

Pseudo-capacitance is the middle part of the battery and electrical double-layer capacitors (EDLCs) (Jiang and Liu, 2019) as shown in Figure 1a. In Figure 1a, Liu points out that the current generation of batteries depends on the Faradic electron transfer from the surface to the metal center based on the charge-compensating ions by intercalation or adsorption. In contrast, a pseudo-capacitor is different from EDLCs because it is not electrostatic-induced and the transfer process of surface electrons distinguishes the behavior from batteries. Pseudo-capacitance can be classified into two categories—intrinsic and extrinsic. The former (Chao et al., 2016) describes an inherent feature of specific materials such as RuO_2_ and MnO_2_, which is on the strength of Faradaic electron transfer. However, the latter emphasizes the technological means of low dimension, nanoscale size, and high surface area (Wang et al., 2007; Brezesinski et al., 2009; Muller et al., 2015; Cook et al., 2016) among a majority of materials, for instance, the single-walled carbon nanotube (SWCNT) with surface-enriched potassium ions in Figure 1b (Hersam, 2009; Kang et al., 2013), attributed to the regular hexagonal arrangement of carbon atoms on the surface. This non-Faradaic pathway provides a possibility to utilize inexpensive carbonaceous anodes (Gogotsi and Penner, 2018) if proper surface treatments such as activization, doping, and plasma processing have been undergone (Chao et al., 2018).

These surface-dominated anodes uptake and absorb potassium ions with fast reaction kinetics during the electrochemistry process, avoiding the hindrance in the intercalation mechanism, which is regarded as the primary cause for high rate property and large capacity of PIBs undergoing charging and discharging processes (Shin et al., 2011; Shao et al., 2013; Chen et al., 2016; Long et al., 2016). As a consequence, this mini-review analyzes the methods for distinguishing the proportion of capacity contribution and summarizes the application of pseudo-capacitance to PIBs very recently, aiming to design a practical performance improvement approach.

## Analyses And Applications Of Pseudo-Capacitance

Generally, it is widely admitted that pseudo-capacitance is not a pure Faradaic progress but a rapid reversible surface redox reaction involved in EDLCs. The charge storage mechanism of complex PIBs behaviors is composed of two typical contribution progresses: surface-induced pseudo-capacitor process and diffusion-dominated process (Brezesinski et al., 2009; Wen et al., 2015; Xu et al., 2019b). Nonetheless, Faradaic and non-Faradaic reactions are electro indistinguishable for jointly contributing to the current parameter (Gogotsi and Penner, 2018). Most researchers employ cyclic voltammetry (CV) to determine the relative proportion of contribution from pseudo-capacitor and diffusion-dominated processes. The peak current is proportional to the square root of sweep rate describing the reversible diffusion-limited state (*i*-$v^{\frac{1}{2}}$), whereas it is proportional to the sweep rate (*i-v*) describing the capacitive state. A representative power law relationship between the current and scan rate reveals the charge storage mechanism in PIBs (Wang et al., 2007; Torsten et al., 2010; Veronica et al., 2013):

$$\begin{array}{l} \text{i}=\text{a}v^{b} \end{array}$$

where *a* and *b* are constants. The *b* value can be figured out by profiling the log(*i*)–log(*v*) curve. If *b* = 0.5, the Faradic diffusion is predominant; while *b* = 1, the pseudo-capacitance assumes the primary contribution (Sathiya et al., 2011; Lijun et al., 2014; Zou et al., 2017). Furthermore, as for a fixed sweep rate, the specific pseudo-capacitance contribution can be given in detail by the following formula (Torsten et al., 2010; Wang Y. et al., 2016):

$$\begin{array}{l} \text{i}=k_{1}v+k_{2}v^{\frac{1}{2}} \end{array}$$

where the parameter *k*_1_*v* represents the capacitive process while the $k_{2}v^{\frac{1}{2}}$ is in favor of the diffusion process as stated earlier.

Furthermore, Marveh maintains that compared with the CV method, the step potential electrochemical spectroscopy (SPECS) has wider adaptive range with prominent advantages. In high sweep rates, SPECS presents precise characterization to depict the process of electrical double layer on the surface of electrodes (Forghani and Donne, 2018).

Recent works validate the validity of the pseudo-capacitance algorithm based on the surface-dominated pseudo-capacitance mechanism, which has been extensively applied in carbonaceous anodes in PIBs by constructing high surface area or activating.

Doping and activating are highly feasible methods that introduce abundant defects, expand specific surface area, and promote the conductivity, meanwhile adding charge storage for PIBs (Lijun et al., 2014; Share et al., 2016; Chen et al., 2017; Lei et al., 2018; Xu et al., 2018).

**Figure 2:**
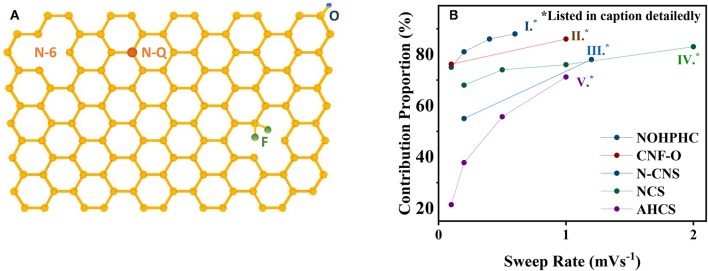
**(A)** Surface defects induced by N-doping, O-doping and F-doping. **(B)** Relationship between pseudo-capacitance contribution proportion and sweep rate based on recent researches (I) (Yang et al., 2018), (II) (Adams et al., 2017), (III) (Lei et al., 2018), (IV) (Chen et al., 2017), (V) (Wang et al., 2018).

Nitrogen-doped strategy has a practical significance eliciting satisfying performance enhancements. According to the X-ray photoelectron spectroscopy (XPS) results, pyrrole nitrogen (N-5), pyridine nitrogen (N-6), and quaternary N (N-Q) are three N-doping forms presented in Figure 2A, where N-5 and N-6 possess high electrochemical activity and generate additional defects in the surface of the graphene layer, hence promoting the adsorption quantity of potassium ions, accelerating the kinetic process (Li et al., 2013; Wang et al., 2014; Xu et al., 2018). This differentiation of N forms is ascribed to their constructions of respective bonding electrons, resulting in different chemical activities. However, N-Q, located in the internal surface of graphene layer, bonding with three sp2 carbon atoms, is beneficial to improve electrical conductivity (Yang et al., 2018). Notably, N-6 is regarded as the most effective doping precursor because it replaces the carbon atom with a nitrogen atom at the defect or the edge of the graphite plane and occupies abundant active centers to adsorb potassium ions (Ma et al., 2012; Ding et al., 2014; Xie et al., 2017). Consistently, recent researches demonstrated that N-6 defects decreases with temperature increasing; meanwhile, the degree of graphitization rises, accompanied by the generation of N-Q. Xu concludes that among three temperature-controlled materials NCNF-650, NCNF-950, and NCNF-1100 derived from poly-pyrrole nanofibers, the pseudo-capacitance contribution of NCNF-650 occupies 90% at 1 mV s^−1^ for abundant N-6 defects, while the others occupy 73 and 84% at 1 mV s^−1^ as displayed in Figure 1c with their *b* value (Xu et al., 2018). The *b* value increases with the temperature dropping, revealing the degree deepening of pseudo-capacity, in accordance with the quantity N-6 defects. Similar results are obtained in Xie's report; nonetheless, Xie indicates that enhancement of electrochemical performance is a comprehensive result associated with N-6 defects, electrical conductivity, and transfer resistance. Three sets of temperature-controlled experiments point out that PNCM-700 is equipped with the best comprehensive performance compared with PNCM-500 and PNCM-900. As for the function of defects, Clement states that the D-bond from the Raman spectrum is employed to describe the sp^3^ defect distribution, which intensifies a six-fold rate performance to the un-doped material (Clement et al., 2015; Share et al., 2016). The prominent significance of N-doping is to spread out the interlayer spacing and provide huge specific surface area to promote the pseudo-capacitive effect.

Doping some other elements also achieves fair results. Oxidation functional groups on the carbon surface polishes up the wettability of carbon-based materials and advances the pseudo-capacitance behavior (Tarun et al., 2010; Shao et al., 2013; Wang X. et al., 2016; Wu et al., 2016; Xie et al., 2017; Wang et al., 2018). Adams reported that oxidation groups increase obviously on the surface capacitive storage while inducing the capacity reduction contributed by the intercalation mechanism. As a consequence, there is no significant enhancement to the total capacity (Adams et al., 2017). In addition, mixed-doping P and O doping (Ma et al., 2017) based on the triphenylphosphine precursor obtains a satisfactory capacity of 474 mA h g^−1^, benefiting from expanding the interlayer spacing; N and F doping immensely adds the conductivity distinctly (Ju et al., 2016; Share et al., 2016; Adams et al., 2017).

Activated hollow carbon nanospheres (HCS) underwent HF etching from C@SiO2 nanospheres in Wang's work. Wang emphasizes on the sharp increase on the surface area from 481.4 to 757.8 m^2^ g^−1^ after activating utilizing KOH as the activator. Capacitive contribution occupies 71.2% at a sweep rate of 1 mV s^−1^, leading to 192.7 mA h g^−1^ at 2 A g^−1^ after 5,000 cycles with a retention of 99.5% (Wang et al., 2018). Aforesaid data support the rule that the pseudo-capacity contribution has the tendency of positive correlation with sweep rate as summarized in Figure 2B. This work claimed that activated hollow carbon expands the layer spacing of the carbon anode and shortens the diffusion distance of K-ions.

Nevertheless, surface-modified strategies, whether doping or activating, may give rise to the decrease of initial Coulombic efficiency (ICE) unsatisfactorily. Compensatory methods work well in LIBs and SIBs (Suo et al., 2017) to establish appropriate binder–electrolyte systems (BESs), which directly impact the formation of solid electrolyte interphase (SEI), especially the morphology features such as thickness, pore, and wrinkle. Tailored BESs shape the SEI into a smooth and thin layer, hence improving the transfer efficiency of ions on the phase interface (Xu et al., 2019a). Similarly, employing KFSI and KTFSI electrolyte (Eftekhari et al., 2016; Jin et al., 2016), adding electrolyte additives (Wu et al., 2017), selecting hydrophilic binders such as CMC, PANa, and SA (Komaba et al., 2015; Luo et al., 2015b; Jin et al., 2016; Xu et al., 2019a), and utilizing pre-potassiation technique (Yang et al., 2018) serve the same purpose for superb PIBs.

## Conclusions And Perspectives

PIBs with carbonaceous anodes provide the possibility for industrialization under controlled price. Facilely, surface modifications such as doping and activating obviously enhance the pseudo-capacitance contribution, speeding up the rate and power performance based on a rapid electrochemical kinetics.

This non-insertion charge storage (pseudo-capacitance absorption) integrates with both battery-type and capacitor-type characteristics, exhibiting distinct redox separation peaks including analogous linear capacitive voltage response. However, the relationship between capacitive and sweep rate is only authentic limited in a low and narrow sweep rate under the CV separation method. Precisely, the SPECS is suitable for a wider range of sweep rates, inducing detailed contribution information for each potential point (Forghani and Donne, 2018). In addition, matching binder–electrolyte with anodes accurately can synergistically promote specific capacity and rate properties, deriving high-performance PIBs.

## Author Contributions

All authors listed have made a substantial, direct and intellectual contribution to the work, and approved it for publication.

### Conflict of Interest

The authors declare that the research was conducted in the absence of any commercial or financial relationships that could be construed as a potential conflict of interest.
